# Food and time: dietary plasticity of different sources of a generalist insect herbivore

**DOI:** 10.1093/jisesa/ieae024

**Published:** 2024-03-28

**Authors:** Katherine Hernandez, M Deane Bowers

**Affiliations:** Department of Ecology and Evolutionary Biology, University of Colorado, Boulder, CO, USA; Department of Ecology and Evolutionary Biology, University of Colorado, Boulder, CO, USA; Museum of Natural History, University of Colorado, Boulder, CO, USA

**Keywords:** generalist, painted lady, host plant, larval performance, immune response

## Abstract

Painted lady butterflies (*Vanessa cardui* L., Nymphalidae) are generalist herbivores and serve as a model system across several fields of biology. While it has been demonstrated that *V. cardui* caterpillars can develop on different host plants, much of this work has been done on commercially sourced caterpillars, which could limit our understanding of wild *V. cardui* populations. In this study, we sought to explore possible differences in how commercial and wild *V. cardui* caterpillars may respond to feeding on different host plants, and subsequently, how their diet impacts immune response and survival. Here, we analyzed performance, survival, and immune response of wild and commercially sourced *V. cardui* caterpillars over several generations on diets that consisted of either 1 of 4 different host plant species or a mixed diet including all 4 species. Qualitatively, we observed that wild larvae had a better larval performance and hemocyte counts compared to the commercial larvae. The results demonstrate that both wild and commercially sourced caterpillars grew and survived best on the same diet treatments (mallow, narrowleaf plantain, and a mixed diet) during development across generations. Immune responses showed similar patterns across host plants between wild and commercial populations, with individuals showing lowered immune responses on dandelion and lupine and higher ones on mallow, plantain and the mixed diet; although the relative rankings on those 3 diets varied. Survival also demonstrated similar patterns, in that individuals reared on dandelion and lupine had the lowest survival.

## Introduction

Herbivorous insects show a continuum in diet breadth, from extreme specialists, which feed on 1 or a very few closely related plant species, to extreme generalists, which feed on hundreds of plant species across dozens of plant families (Bernays 1991, Ali and Agrawal 2012). Insects considered generalists, however, may be generalists in different ways. For example, different populations of a generalist species may actually be relatively specialized (Fox and Morrow 1981). Alternatively, individual insects of a generalist species may feed on many different host plants during their lifetime (Bernays and Minkenberg 1997, Singer et al. 2002). In some species, individual females may oviposit on different host plant species; therefore, offspring could develop on host plants that differ from those of their parents and other offspring of the same female (Obermaier et al. 2008, Garcia-Robledo and Horvitz 2012). For any of these generalist strategies, the host plant species used may also influence interactions with higher trophic levels. For example, some host plant species may provide sequesterable defense compounds that aid in insect defense, while others do not (Lampert and Bowers 2010, 2015, Knerl and Bowers 2013). Diet may also influence the caterpillar immune response, which targets enemies such as parasitoids and pathogens (Lampert 2010, Muchoney et al. 2022).

Although generalists may feed on many different plant species, there is often variation in their preference and performance on those different plant species (Bernays et al. 1994, Bernays and Minkenberg 1997, Unisicker et al. 2008). In addition, the availability and suitability of particular plant species may change during a growing season (Stewart et al. 2021), which in turn can impact herbivore distribution as they search for suitable host plants over the course of the season(s) (Bernays et al. 1994, Stephens and Myers 2012, Darwell et al. 2014). The variation in host plant availability and suitability and the environments in which these plants occur can provide challenges to multivoltine herbivores, in which different generations may encounter distinct suites of environmental conditions, host plants, and natural enemies (Ostergard and Ehrlen 2005, Massad and Dyer 2010, Ali and Agrawal 2012).

Higher trophic levels may also be influenced by the plant on which an insect herbivore feeds (Price et al. 1980, Shikano 2017). Generalist insect herbivores can mitigate some of the negative impacts imposed by natural enemies by switching host plants during development, which can allow opportunities to feed on higher quality plants and/or plants that may have defensive compounds that act as defenses against their enemies (Richards et al. 2010, Singer et al. 2014). A critical part of an insect’s defense system involves its ability to mount an immune response (Coley et al. 2006, Cory and Hoover 2006, Klemola et al. 2007). However, there may be trade-offs to mounting an immune response, such as slower growth rates and increased development times (Rivero et al. 2001, Cotter et al. 2003, Freitak et al. 2003, Schmid-Hempel 2005, Diamond and Kingsolver 2010, Ardia et al. 2012). Slowed larval development means more time spent exposed to enemies and increased opportunities for additional attacks by other organisms (Havill and Raffa 2000).

The cosmopolitan Painted Lady butterfly (*Vanessa cardui*, Nymphalidae) is unusual among butterfly species in that it has a very broad diet breadth, reported to feed on host plants from 25 families (Scott 1986, Janz 2005). Females oviposit their eggs singly and an individual female typically uses different host plant species for oviposition (O’Neill et al. 2010). *Vanessa cardui* is a migratory species that has one of the largest distributions of terrestrial animals undertaking large-scale migrations (Shields 1992, Talavera and Vila 2017). This species does not undergo diapause and successive generations migrate year-round (Talavera and Vila 2017). Because of its broad geographic range, dietary breadth, and multivoltine life cycle, *V. cardu*i serves as an important model system for studies on the ecology and evolution of generalist insect herbivores (Monteiro et al. 1994, Stefanescu et al. 2007, 2016, Brattström et al. 2008, O’Neill et al. 2010).

Much of what we know about *V. cardui* development and behavior in North America comes from studies that use commercial stock of these butterflies (e.g., Monteiro et al. 1994, Serfas and Carroll 2005, Connahs et al. 2016). Commercially available insect herbivores are easily accessible, but are typically reared under very controlled conditions, often on artificial diets, and may suffer from inbreeding depression, and experience laboratory adaptations (Boller and Chambers 1977, Hoffman and Ross 2018). To understand how the source of a study species, whether it is from wild or commercial sources, might affect aspects of their biology, a number of studies have made direct comparisons between commercially sourced and wild collected strains (see Bowers and Puttick 1989, Kingsolver 2007, Hoffman and Ross 2018, Lee et al. 2022). For example, if insects are reared on artificial diets, their performance on different host plant species may be quite different from their naturally occurring counterparts. A number of studies have used laboratory strains to examine growth, development, and immunity (Stefanescu et al. 2007, O’Neill et al. 2010, Connahs et al. 2016). Given the extensive use of laboratory strains of many well-studied insect herbivores, the goal of this research was to compare the performance and immunocompetence of a laboratory strain of *V. cardui* with those of a wild strain of *V. cardui,* when larvae were reared on different host plants over several generations.

We used larvae of *V. cardui* to ask questions about the role of diet in mediating larval performance and immune response and how these responses might change over multiple generations for both commercial and wild collected strains: (Q1) How do diet and generation affect larval performance when reared on different host plant species or a mixed diet? We expected wild sourced caterpillars to perform better on host plants across generations because they are more adapted to a natural diet as compared to individuals from a lab culture. We also expected caterpillars from both sources to perform better on a mixed diet compared to a single diet because *V. cardui* is a known generalist herbivore. (Q2) Are there differences in the immune response based on diet and does the immune response vary across different generations? Wild sourced caterpillars would be expected to mount a stronger immune response as a result of adaptation to host plants and having to deal with natural enemies, whereas laboratory cultured individuals would be expected to mount a weaker immune response due to the lack of natural enemies in their environment.

## Materials and Methods

### Study Species

*Vanessa cardui* is a generalist herbivore and long-distance migrant (Stefanescu et al. 2016). This species is one of the most widespread of all butterflies, occurring on every continent with the exception of Antarctica (Wahlberg and Rubinoff 2011). Most scientific research on *V. cardui* has focused on their migratory behavior (Stefanescu et al. 2007, Brattström et al. 2008), ecology (Ellis and Bowers 1998, O’Neill et al. 2010), and wing pattern development (Monteiro et al. 1994, Serfas and Carroll 2005, Connahs et al. 2016). This long-distance migrant can encounter different environments, host plants, natural enemies, and climates over the course of multiple generations, and its feeding plasticity may play a role in its ability to succeed in very different habitats.

### General Experimental Design

To examine the effects of diet and time (i.e., generation) on performance and immune response of commercial and wild populations of *Vanessa cardui*, 2 separate experiments were conducted in successive years (2017 and 2018) using 2 different sources of butterflies with which to start experimental lines. The 2017 experiment used eggs sourced from Carolina Biological Supply (North Carolina, USA) (hereafter referred to as commercial), which were reared over 4 successive generations (initial *N* = 200/generation; 40 per diet on 5 diets; total initial *N* = 800). The experiment conducted in 2018 used eggs sourced from wild butterflies collected in Boulder County, Colorado (hereafter referred to as wild), which were reared over 3 successive generations (initial *N* = 200/generation; total initial *N* = 600). Adults from caterpillars reared on each of the diets were placed in mesh tents (BugDorm 2120 Insect Rearing Tent; 60 × 60 × 60 cm) to breed the next generation, with all individuals in a tent coming from the same diet treatment. Leaves from 4 host plant species were used to feed caterpillars from both sources (see below) and were collected repeatedly from locally growing plants at several sites in Boulder County, USA. Plants were collected from natural populations in order to capture ontogenetic changes in host plants that occur over the growing season. For each experiment, there were 5 treatment groups, 4 of which subjected caterpillars to a single host plant species over the course of their development and a fifth in which caterpillars were fed a mixed diet of all 4 plant species (see below).

To obtain larvae for the experiments, for each generation, butterflies were mated in mesh tents located in a greenhouse. The butterflies were separated by diet treatment and kept isolated in their respective tents for mating, which was allowed to occur only between individuals from the same diet treatment. To standardize the substrate for oviposition, all females were given 1 of the host plant species, *Plantago lanceolata* (Plantaginaceae); individual plants for oviposition were chosen at random from an existing greenhouse population. The eggs were then collected off the plant by hand using number 3 paintbrushes (creative inspirations) and moved to the appropriate host plant. Caterpillars coming from butterflies that were fed a specific diet were also fed on the same diet during their development. This process was repeated for all generations of the experiment.

### Host Plants

The experiments focused on 4 host plant species: narrowleaf plantain, *Plantago lanceolata* (Plantaginaceae), common dandelion, *Taraxacum officinale* (Asteraceae), common mallow, *Malva neglecta* (Malvaceae), and silvery lupine, *Lupinus argenteus* (Fabaceae). Narrowleaf plantain, common mallow, and common dandelion were introduced from Europe and silvery lupine is a native plant of North America (USDA PLANTS Database). Narrowleaf plantain, common dandelion, and common mallow have a wide distribution across the North America, and the distribution of silvery lupine is confined to the western half of North America (Kartesz 2015). These 4 host plants were chosen for this study because they all coexist with the painted lady butterfly’s range across the Front Range of Colorado and have been reported as host plants (Scott 1986, Janz and Nylin 1997, Ackery 1988). The fifth diet treatment consisted of a mixture of fresh leaves from these 4 plant species that was provided to each individual caterpillar.

### Larval Performance

Growth rates, pupal weights, and survival were recorded as measures of performance for both wild and commercial larvae. We reared all caterpillars individually in closed 4-ounce Reditainer plastic containers at ambient room temperature (approx. 22 °C) and the natural daylength that changed over the course of the season. Leaves were replaced with fresh ones at least every other day for every individual caterpillar, ensuring that caterpillars never ran out of food. We began weighing the larvae when they molted into their third instar and then weighed them every 48 h with their final weight recorded after they molted to the fifth instar. These data were used to calculate relative growth rate (RGR) as follows: RGR = (larval weight gain)/ (average larval weight during trial) * (number of days) (Waldbauer 1968). Pupae were weighed 2 days after pupation and survival was to the pupal stage.

### Immune Response

We measured 2 components of the immune response for both commercial and wild caterpillars on each diet treatment: hemocyte count and encapsulation (Triggs and Knell 2012, Smilanich et al. 2018). Hemocytes are considered to be the primary cellular response of the insect immune systems (Lavine and Strand 2002, Strand, 2008). Encapsulation is a process in which hemocytes within an insect’s body attach to foreign bodies (such as parasitoids or pathogens), ultimately melanizing and killing the invader by asphyxiation and the production of cytotoxic compounds (Strand 2008). The strength of the encapsulation response can be measured by quantifying melanization on fine filaments or tiny beads that are inserted into the insect’s body (Nappi and Christensen 2005); we used nylon filaments (Carper et al. 2019). Immune response was measured for the first and last generations of commercial caterpillars and for all 3 generations of wild caterpillars.

To determine hemocyte count and encapsulation response, newly molted fifth-instar larvae from each diet treatment (*N* = 10/diet treatment) were weighed, then were placed in a freezer (−29 °C) for approximately 1 min to slow caterpillar movement during the assay (Carper et al. 2019). Each caterpillar was then moved to a watch glass and a fine (00) insect pin was used to make a small hole behind the 4th proleg in each caterpillar. To quantify hemocytes, 10 µl of hemolymph was removed with a pipette after making the puncture. The 10 µl of hemolymph was added to 10 µl of anticoagulant and stored on ice. We prepared anticoagulant using a mixture of EDTA, citric acid, and a phosphate-buffered saline (as in Smilanich et al. 2018). We took a 10 µl aliquot of hemolymph-anticoagulant mixture and counted the hemocytes with a hemocytometer (Sigma-Aldrich Bright-Line) under a compound microscope and calculated cell density per ml of hemolymph following Triggs and Knell (2012) protocols.

To measure melanization, we inserted nylon filaments into caterpillars immediately after taking the hemolymph sample. The filaments were made using 0.20 mm in diameter monofilament fishing line (Berkley Trilene XL Smooth Casting), following Carper et al. (2019). The fishing line was lightly sanded using sanding paper and trimmed to 2 mm, with either a knot tied or heated at 1 end with a lighter to melt and create a blunt head on 1 side. Then the nylon filament was inserted into the puncture created with the insect pin and left there for 24 h. The caterpillars were returned to their containers and allowed to feed on their respective diets during the 24-h period. The filament was then removed, photographed, and Adobe Photoshop (Adobe, Inc.) was used to determine the level of encapsulation for each caterpillar. Photographs were analyzed in Adobe Photoshop by setting to grayscale with 0 as white and 255 as black. The “magic wand” tool was used to select the part of the filament that was inserted into the caterpillars to be measured for encapsulation. The mean white value was calculated using the histogram tool for each filament to get mean white values for each diet (Carper et al. 2019). The mean white values are the average amount of white per pixel across the filament and the inverse of the mean represents the average darkness of the filament, the darker the color the higher the degree of encapsulation on the filament (Smilanich et al. 2009, Carper et al. 2019). We used this mean as the quantitative measure of immune response (Smilanich et al. 2018, Carper et al. 2019).

### Statistical Analysis

All statistical analyses for both experiments were run in R (R Core Team version 4.1.2). Two-way ANOVAs were performed on log transformed data for both sets of experiments to examine how diet, generation, and their interaction affected growth rates and pupal weights across 4 generations of commercial caterpillars (2017) and 3 generations of wild sourced caterpillars (2018). For the 2017 experiment, two-way ANOVAs on log transformed data were used to evaluate how diet, generation, and their interaction affected larval immune response (hemocyte counts and melanization) in the first and fourth generations. The 2018 experiment used two-way ANOVAs on log transformed data to evaluate how diet, generation, and their interaction affected larval immune response (hemocyte counts and melanization) in all 3 generations. The survival data for both experiments were analyzed using a binomial logistic regression in R using the lme4 package (Bates et al. 2015).

## Results

### Larval Performance

#### Wild population.

**Fig. 1.**
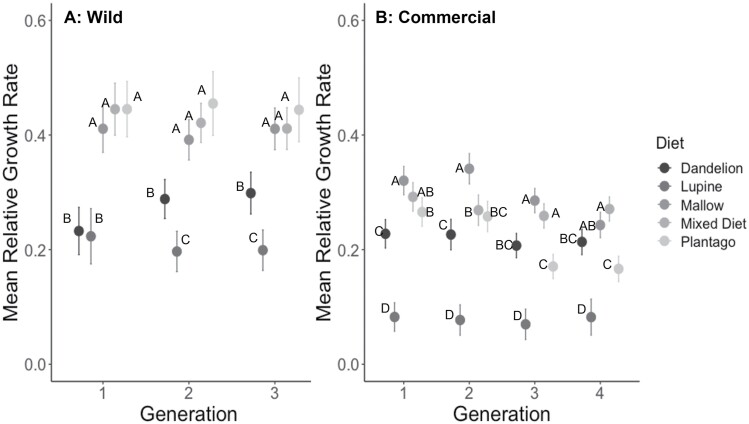
Mean (+SE) relative growth rates from A) 3 generations of wild and B) 4 generations of commercial *V. cardui* larvae that were fed 1 of 5 diet treatments. Means with different letters within a generation are significantly different (Tukey’s HSD, *P* < 0.05).

**Fig. 2.**
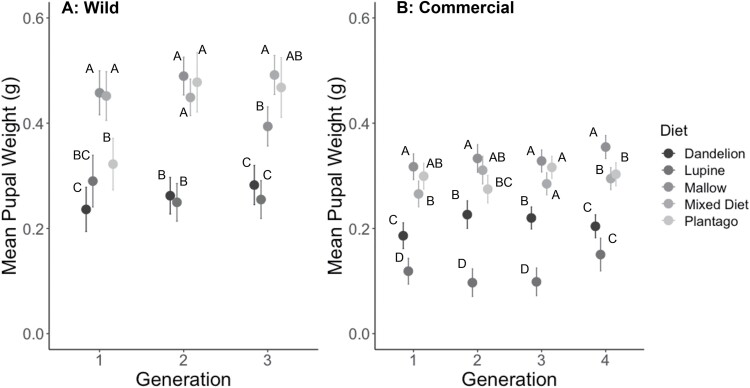
Mean (+SE) pupal weight from A) 3 generations of wild and B) 4 generations of commercial *V. cardui* larvae that were fed 1 of 5 diet treatments. Means with different letters within a generation are significantly different (Tukey’s HSD, *P* < 0.05).

**Fig. 3.**
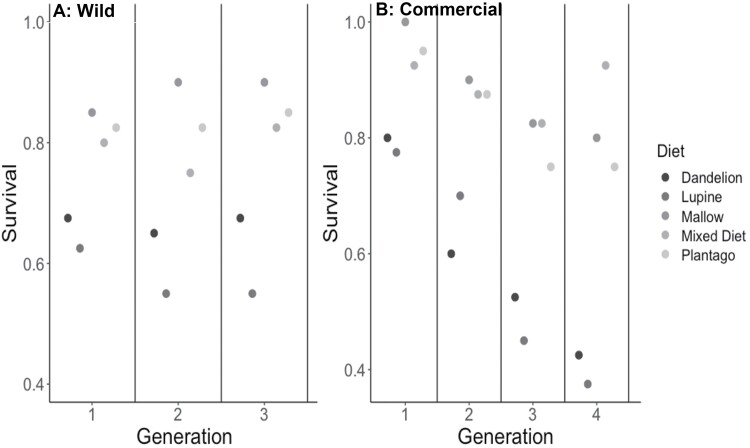
Survival of larvae across all generations and by diet for A) wild sourced larvae and B) commercial sourced larvae.

Growth rates of larvae from the wild population (Fig. 1A) were significantly affected by host plant, but not by generation and there was no interaction between diet and generation (Table 1). Post hoc Tukey tests on diet showed that larvae fed on lupine and dandelion had slower growth rates than those fed the other 3 diets in each generation (Fig. 1A). Pupal weights (Fig. 2A) differed significantly with diet, but did not differ by generation; however, there was a significant interaction between diet and generation (Table 1), indicating that the effect of diet on pupal weight changed over the course of the 3 generations. Specifically, post hoc Tukey tests on diet in each generation showed that larvae fed on lupine, dandelion, and narrowleaf plantain had lower pupal weights than those fed on the mallow and mixed diets in generations 1 and 3, but pupal weight of larvae fed narrowleaf plantain in generation 2 were not different from those that fed on mallow and the mixed diet (Fig. 2A). The survival (Fig. 3A) of larvae differed by diet and generation and there was not a significant interaction of diet and generation (Table 2).

**Table 1.** Statististical results for *V. cardui* larval performance

	Growth rate		Pupal weight		Melanization		Hemocyte count	
	*F* statistic	*P*	*F* statistic	*P*	*F* statistic	*P*	*F* statistic	*P*
Wild								
Diet	*F*_(4,168)_ = 81.9	***	*F*_(4,157)_ = 74.47	***	*F*_(4,91)_ = 135.5	***	*F*_(4,91)_ = 145.7	***
Generation	*F*_(1,168)_ = 0.099	*P* = 0.754	*F*_(1,157)_ = 0.535	*P* = 0.465	*F*_(1,91)_ = 2.01	*P* = 0.153	*F*_(1,91)_ = 0.458	*P* = 0.499
Diet*Generation	*F*_(4,168)_ = 1.76	*P* = 0.138	*F*_(4,157)_ = 6.25	***	*F*_(8,91_ = 1.85	*P* = 0.121	*F*_(4,91)_ = 0.256	*P* = 0.906
Commercial								
Diet	*F*_(4,442)_ = 154.4	***	*F*_(4,384)_ = 177.3	***	*F*_(4,109)_ = 116.1	***	*F*_(4,109)_ = 394.1	***
Generation	*F*_(1,442)_ = 45.57	***	*F*_(1,384)_ = 6.99	**	*F*_(1,109)_ = 0.456	*P* = 0.502	*F*_(1,109)_ = 0.222	*P* = 0.639
Diet*Generation	*F*_(4,442)_ = 8.10	***	*F*_(4,384)_ = 0.208	*P* = 0.933	*F*_(4,109)_ = 2.02	*P* = 0.101	*F*_(4,109)_ = 4.54	**

**Table 2.** Statistical results for *V. cardui* survival

Survival	*df*	Deviance	Resid. *df*	Resid. dev	Pr(>Chi)
Wild					
NULL	599	674.80			
Diet	4	40.986	595	633.82	***
Generation	1	0.014	594	633.80	0.905
Diet: Generation	4	1.099	590	632.70	0.894
Commercial					
NULL	799	895.32			
Diet	4	85.828	795	809.49	***
Generation	1	41.457	794	768.03	***
Diet: Generation	4	5.434	790	762.60	0.246

#### Commercial population.

Larval growth rates (Fig. 1B) differed significantly by diet and generation, and there was a significant interaction of diet and generation (Table 1), indicating that the effect of diet on larval growth changed with generation. Specifically, post hoc Tukey tests on diet within each generation showed that larvae fed on lupine had lower growth rates; however, dandelion was the second poorest diet in generations 1 and 2, but narrowleaf plantain was the second poorest diet in generations 3 and 4 (Fig. 1B). Pupal weights (Fig. 2B) differed significantly by diet and generation, and there was a signification interaction of diet and generation (Table 1), indicating that the effect of diet on pupal weight changed with generation. Post-hoc Tukey tests on diet showed that larvae fed on lupine and dandelion had lower pupal weights than those fed on the other 3 diets; however, the rankings of the other 3 diets varied with generation (Fig. 2B). Specifically, the pupal weights of individuals fed on dandelion and lupine showed an increase or a decrease in pupal weights, respectively, across generations. The survival of larvae during development did differ by diet but not by generation and there was no interaction between diet and generation (Fig. 3B).

### Immune Response

#### Wild population.

**Fig. 4.**
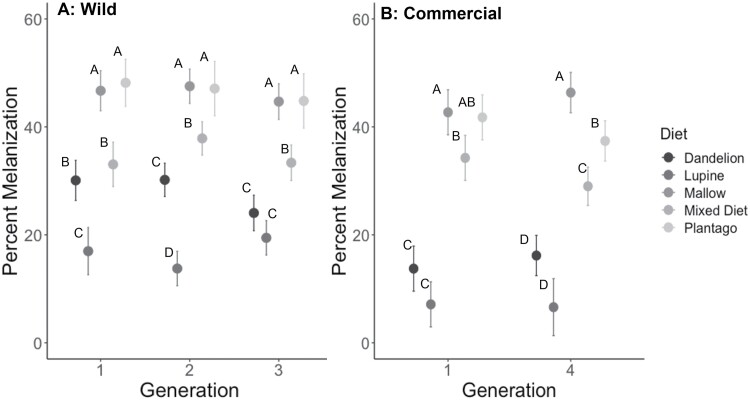
Percent melanization (+SE) from A) 3 generations of wild and B) first and fourth generations of commercial larvae of *V. cardui* larvae that were fed 1 of 5 diet treatments. Within a generation, data points with different letters are significantly different (Tukey’s HSD, *P* < 0.05).

**Fig. 5.**
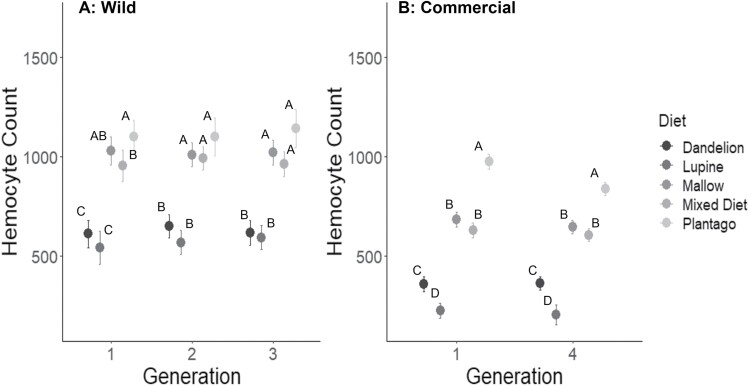
Hemocyte count (+SE) from A) 3 generations of wild and B) first and fourth generations of commercial larvae of *V. cardui* larvae that were fed 1 of 5 diet treatments. Within a generation, data points with different letters are significantly different (Tukey’s HSD, *P* < 0.05).

Melanization (Fig. 4A) differed significantly with diet but not by generation; nor was there a significant interaction of diet and generation (Table 1). Post hoc Tukey tests on diet showed that larvae fed on lupine had lower melanization than those fed the other diets; melanization of larvae fed on dandelion changed slightly in its ranking among the diets, but mallow, and narrowleaf plantain were always the diets producing the highest melanization (Fig. 4A). Hemocyte counts (Fig. 5A) were significantly affected by diet but did not differ by generation, nor was there an interaction of diet and generation (Table 1). Post-hoc Tukey tests on diet showed that larvae fed on lupine and dandelion had lower hemocyte counts than those fed the other 3 diets (Fig. 5A), which did not differ from each other, except in generation 1, in which larvae fed on the mixed diet had significantly lower melanization than those reared on narrowleaf plantain.

#### Commercial population.

Measurements of melanization (Fig. 4B) and hemocyte counts (Fig. 5B) were made for the first and fourth generations to compare immune responses across diet treatments and between the first and last generation. There were not enough larvae to perform immune assays on larvae from generations 2 and 3. Melanization (Fig. 4B) differed significantly with diet but did not significantly differ by generation, nor was there an interaction of diet and generation (Table 1). Post-hoc Tukey tests on diet showed that larvae fed on lupine and dandelion had lower and similar levels of melanization compared to those fed the other diets (Fig. 4B); in the fourth generation larvae fed on mallow, narrowleaf plantain, or mixed diet differed significantly from each other. Hemocyte counts (Fig. 5B) also differed significantly with diet; although there was no effect of generation, there was an interaction of diet and generation, indicating that the response of hemocyte counts to diet changed across generations (Table 1). Post-hoc Tukey tests on diet showed that larvae fed on lupine and dandelion had lower hemocyte counts than those fed the other 3 diets; larvae fed on mallow and the mixed diet were intermediate and not significantly different from each other, while larvae fed on narrowleaf plantain had the highest melanization (Fig. 5B).

## Discussion

Overall, our results showed that for larvae from both wild and commercial populations of this generalist insect herbivore, lupine and dandelion were inferior host plants, resulting in reduced growth, pupal weight, survival, and immune response. Although we were unable to directly compare the data for the wild and commercial populations, there were some notable qualitative differences: (i) Relative growth rates were lower for commercial larvae, ranging from means of 0.1–0.4, whereas wild larvae showed higher growth rates ranging from 0.2 to 0.5 (Fig. 1). (ii) Pupal weights were lower for commercial larvae, ranging from 0.1 to 0.4 g, compared to those of wild larvae, which ranged from 0.2 to 0.55 g (Fig. 2). (iii) Hemocyte counts were generally lower for the commercial larvae, ranging from 200 to 1,000, compared to the wild larvae, which ranged from 500 to 1,200 (Fig. 5), although melanization did not appear to differ (Fig. 4). (iv) Differences across generations were not apparent in the wild larvae, although the commercial larvae showed changes across generations for growth rate and pupal weight (Table 1). It is also important to note that experiments done on commercial and wild larvae occurred in different years: experiments on commercial larvae were carried out in the summer of 2017 and those for the wild larvae in summer 2018.

Although larvae from commercial rearing sources are often used for experimental purposes (e.g., Kingsolver 2007, Liedo et al. 2007, Hoffmann and Ross 2018), our understanding of how the origins of larvae (wild compared to laboratory cultures) might affect the results of experiments that use them is confined to relative few species (Hoffman and Ross 2018). Commercial cultures of the painted lady are commonly used to examine many different questions about the ecology and evolution of butterflies (e.g., Monteiro et al. 1994, O’Neill et al. 2010, Connahs et al. 2016, Zhang et al. 2021), and this study is the first to investigate how wild and commercial populations of this model generalist species might compare in their response to different diets. A number of studies have directly compared laboratory and wild strains of other species and found important differences (reviewed in Hoffmann and Ross 2018). Additional lepidopteran examples include Kingsolver (2007), which found that growth rates of *Manduca sexta* (Sphingidae) differed between wild and commercial strains and that individuals from the commercial strain grew faster when larvae were reared on the same diets and under the same conditions. There was also more variation in the growth rates of larvae in the wild colony, indicating more developmental plasticity in the wild population (Kingsolver 2007). Another study examined the effect of dietary iridoid glycosides on laboratory and wild strains of *Lymantria dispar* (Lymantriidae) (Bowers and Puttick 1989) and found that larvae from a wild population showed decreased weights when fed increased levels of the iridoid glycoside catalposide, but larvae from a laboratory strain showed no dose-dependent effect of this compound. A very recent study found differences in the behavior of wild and domesticated *Lymantria dispar dispar* (Lymantriidae) in response to the predation risk of a paper wasp (*Mischocyttarus* sp.) and showed that larvae from the domesticated population showed no response to wasps, whereas wild larvae did (Lee 2022). These studies show that larvae from wild and laboratory strains can differ in their responses to experimental conditions and suggest that consideration should be given to the origin of insects used in experiments, encouraging caution in how we extrapolate results of experiments using lab-reared insects (Lee 2022).

Insect herbivores deal with many challenges during their development, but studies show that the plants on which they feed are likely the most important (Tikkanen et al. 2000, Mody et al. 2007, Salgado and Saastamoinen 2019) and results from our experiments with both the wild and commercial populations are no exception. We found that diet significantly affected performance in larvae from both wild and commercial populations. Similar variation in the suitability of different host plant taxa have been found in many other generalist insects. For example, *Trichoplusia ni* (Noctuidae) caterpillars fed on 6 different diets to examine their performance consistently performed best on 3 of the 6 host plants during their development (Shikano 2017). Similarly, when looking at grape cultivars, Moreau et al. (2006) found that development time of the European grapevine moth, *Lobesia botrana* (Tortricidae) varied depending on the cultivar on which they fed. Additionally, similar variation in host plant suitability was found in fall webworm, *Hyphantria cunea* (Erebidae), where individuals fed on narrowleaf cottonwood and chokeberry developed faster and had higher pupal weights compared to those on crabapple and alder (Murphy and Loewy 2015). These are only a few of the many examples of such effects, which are widespread among generalist insect herbivores (Price et al. 1980, Koricheva et al. 1998).

The results of the experiments described here show that, in addition to its effect on performance, diet also affected immune responses for both wild and commercial caterpillars. Furthermore, the diets that resulted in the poorest larval performance (dandelion and lupine) also elicited the lowest immune responses. Similar effects of diet on immune response have been shown in other studies; for example, in the fall webworm (*Hyphantria cunea,* Erebidae), diets that resulted in poor larval performance also resulted in reduced caterpillar immune response (Vyas and Murphy 2022). In another study using a generalist agricultural pest, diet had an impact on the immune response of *Spodoptera littoralis* (Noctuidae) caterpillars (Green 2021): individuals fed on cabbage or cotton showed enhanced immune function compared to individuals that fed on maize. In the Baltimore checkerspot butterfly (*Euphydryas phaeton,* Nymphalidae), larvae fed on an introduced host plant, *Plantago lanceolata* (Plantaginaceae), had lower immune function than those that fed on the native plant *Chelone glabra* (Plantaginaceae) (Muchoney et al. 2022). These studies, coupled with our results, demonstrate that diet is a critical component of the ability of herbivorous insects to mount an immune response.

In our study, larval responses to diet were examined across multiple generations, and the effect of generation differed between larvae from wild versus commercial cultures. Specifically, generation was not a significant source of variation in wild larvae for either performance or immune response; however, there was a significant effect of generation on growth rate and pupal weight for commercial larvae. Although relatively few generations were examined in the present study, other studies have demonstrated changes in larval responses in just a few generations. For example, Wu et al. (2006) found that when fed on a low-quality diet (wheat), the third-generation cotton bollworm (*Helicoverpa armigera* Erebidae) showed longer development times compared to the first and second generations. In 1 of the earlier studies, using tobacco budworm, *Heliothis virescens* (Erebidae), changes in mating behavior were observed after a wild collected colony had spent only 6 generations in the laboratory (Raulston 1975). In another study involving cowpea weevils (*Callosobruchus maculatus*) fed on either azuki beans (*Phaseolus radiatus*) or pigeon peas (*Cajanus cajan*), the development times of the weevils on each host were not significantly different within a generation, but trends over 11 generations suggested differences across generations in their development times (Wasserman and Futuyma 1981). These findings along with our results demonstrate that patterns and/or significant effects can be found in the responses of insect herbivores across generations and in a relatively short amount of time.

The results found in this study provide important information for better understanding the differences between wild and commercial larvae. Our findings showed qualitative differences between the 2 sources of painted lady caterpillars, with commercial larvae having lower performance and 1 measure of immunocompetence, hemocyte counts, than wild larvae. These differences highlight the need to consider where we source experimental insects, such as the painted lady, that are often used in research because of their availability and ease of culture (O’Neill et al. 2010, Connahs et al. 2016, Zhang et al. 2021). Commercially reared organisms are the result of domestication because of artificial selection and potential inbreeding within those populations, which could alter their responses compared to their wild counterparts when addressing questions pertinent to natural systems (Hoffmann and Ross 2018, Perez et al. 2021). Results of this study support those on other insect species and emphasize the need to be cautious in how we extrapolate results of experiments using lab-reared insects.
